# Draft Genome Sequences of Salmonella enterica subsp. *enterica* Serotype Heidelberg from Chicken and Turkey Farm Environments

**DOI:** 10.1128/MRA.01204-18

**Published:** 2018-11-21

**Authors:** Loïc Deblais, Joy Scaria, Gireesh Rajashekara

**Affiliations:** a Food Animal Health Research Program, Department of Veterinary Preventive Medicine, The Ohio State University, OARDC, Wooster, Ohio, USA; b Department of Plant Pathology, The Ohio State University, OARDC, Wooster, Ohio, USA; c Department of Veterinary and Biomedical Sciences, South Dakota State University, Brookings, South Dakota, USA; University of Maryland School of Medicine

## Abstract

Thirty-one different Salmonella enterica subsp. *enterica* serotype Heidelberg isolates collected from several chicken- and turkey-associated farm environments in the Midwestern United States were analyzed using whole-genome sequencing. Availability of these genome sequences may shed light on *Salmonella* adaptation to different farm environments.

## Abstract (precis)

Thirty-one different Salmonella enterica subsp. *enterica* serotype Heidelberg isolates collected from several chicken- and turkey-associated farm environments in the Midwestern United States were analyzed using whole-genome sequencing.

## ANNOUNCEMENT

Nontyphoidal *Salmonella* is one of the leading causes of human foodborne gastroenteritis in North America, with public health costs of approximately $695 million annually in the United States (1). According to the National Outbreak Reporting System (NORS) database (Centers for Disease Control and Prevention), 38% of all salmonellosis outbreaks recorded in the United States between 2012 and 2016 were associated with poultry (https://wwwn.cdc.gov/norsdashboard/). Salmonella enterica subsp*. enterica* serotype Heidelberg is the most common serotype isolated in all poultry breeder types and throughout all levels of the production chain in the United States and Canada (2–4). Rapid adaptation of Salmonella to the poultry host and the farm environment where the birds are raised reduces the antimicrobial efficacy of control methods implemented by the poultry industry (5). However, little is known about the impact of the farm environment on the genome content of Salmonella spp., which may facilitate adaptation to different environments. To this end, we sequenced the genomes of 31 Salmonella Heidelberg isolated from environmental samples obtained from chicken or turkey farms (6). Nineteen and 12 isolates were collected from environmental booties of 16 chicken and 8 turkey breeder farms, respectively, and 2 isolates were collected from hatchery debris in 2 different turkey farms (Table 1). For environmental bootie samples, fabric shoe covers premoistened with skim milk were used and walked on the floor inside the farm over a distance of 305 m. Samples were collected under the supervision of the Minnesota Board of Animal Health between April and July 2015 as part of the National Poultry Improvement Plan (NPIP) Salmonella monitoring program. Salmonella Heidelberg was isolated on a brilliant green agar with novobiocin, xylose lysine tergitol-4, and Millier-Mallinson medium. The identities of the 31 isolates were further confirmed by a PCR assay using one set of primers specific to the Salmonella genus (for OMPC, ATCGCTGACTTATGCAATCG [forward] and CGGGTTGCGTTATAGGTCTG [reverse]; amplicon length, 204 bp) and another set specific to the Salmonella. Heidelberg serotype (for GenBank accession number ACF69659, TGTTTGGAGCATCATCAGAA [forward] and GCTCAACATAAGGGAAGCAA [reverse]; amplicon length, 216 bp) and compared to a known Salmonella Heidelberg strain (7–9).

**TABLE 1:** Metadata of the 31 *Salmonella enterica* subsp. *enterica* serotype Heidelberg isolates from chicken and turkey farm environments

Isolate	Sample origin	Sample type	NCBI genome accession no.	NCBI SRX accession no.	No. of contigs	Contig *N*_50_^*a*^	GC content (%)	Total no. of sequencing reads
C_NS001	Chicken	Environmental bootie	PGWO00000000	SRX3441541	53	412,084	51.5	948,617
C_NS002	Chicken	Environmental bootie	PGWN00000000	SRX3441539	51	412,084	51.5	1,025,661
C_NS003	Chicken	Environmental bootie	PGWM00000000	SRX3441540	52	412,084	51.8	838,953
C_NS004	Chicken	Environmental bootie	PGWL00000000	SRX3441522	71	412,084	51.8	622,170
C_NS005	Chicken	Environmental bootie	PGWK00000000	SRX3441521	93	412,084	51.7	491,774
C_NS006	Chicken	Environmental bootie	PGWJ00000000	SRX3441528	52	291,725	52.3	715,859
C_NS007	Chicken	Environmental bootie	PGWI00000000	SRX3441527	76	144,340	52.5	906,054
C_NS008	Chicken	Environmental bootie	PGWH00000000	SRX3441530	49	411,223	52.2	1,038,062
C_NS009	Chicken	Environmental bootie	PGWG00000000	SRX3441529	65	291,730	52	1,006,172
C_NS010	Chicken	Environmental bootie	PGWF00000000	SRX3441524	74	184,236	52.2	826,444
C_NS011	Chicken	Environmental bootie	PGWE00000000	SRX3441523	63	276,222	51.4	796,049
C_NS020	Chicken	Environmental bootie	PGWD00000000	SRX3441526	71	412,018	52.9	667,956
C_NS024	Chicken	Environmental bootie	PGWC00000000	SRX3441525	88	203,724	51.5	1,151,538
C_NS025	Chicken	Environmental bootie	PGWB00000000	SRX3441542	58	411,826	51.3	887,634
C_NS026	Chicken	Environmental bootie	PGWA00000000	SRX3441543	53	411,820	52.2	769,371
C_NS027	Chicken	Environmental bootie	PGVZ00000000	SRX3441531	51	411,985	52.2	761,794
C_NS028	Chicken	Environmental bootie	PGVY00000000	SRX3441532	71	235,527	52.1	937,797
C_NS029	Chicken	Environmental bootie	PGVX00000000	SRX3441533	95	170,930	52.1	787,619
C_NS030	Chicken	Environmental bootie	PGVW00000000	SRX3441534	68	291,725	52.2	646,325
T_NS-012	Turkey	Environmental bootie	PGVV00000000	SRX3441535	70	248,810	52.2	884,633
T_NS-013	Turkey	Environmental bootie	PGVU00000000	SRX3441536	56	424,424	52.5	1,043,609
T_NS-014	Turkey	Environmental bootie	PGVT00000000	SRX3441537	78	231,397	52.4	883,270
T_NS-015	Turkey	Environmental bootie	PGVS00000000	SRX3441538	88	231,397	52.4	972,069
T_NS-016	Turkey	Hatchery debris	PGVR00000000	SRX3441519	71	291,727	52.6	1,099,328
T_NS-017	Turkey	Environmental bootie	PGVQ00000000	SRX3441520	72	233,820	51.7	1,128,127
T_NS-018	Turkey	Environmental bootie	PGVP00000000	SRX3441515	60	423,224	52.1	920,022
T_NS-019	Turkey	Hatchery debris	PGVO00000000	SRX3441516	102	298,617	51.9	611,230
T_NS-031	Turkey	Environmental bootie	PGVL00000000	SRX3441511	78	240,110	53	775,776
T_NS-032	Turkey	Environmental bootie	PGVK00000000	SRX3441512	100	175,042	53	1,139,971
T_NS-033	Turkey	Environmental bootie	PGVJ00000000	SRX3441513	94	154,373	52.9	1,511,607
T_NS-034	Turkey	Environmental bootie	PGVI00000000	SRX3441514	153	82,339	52.6	1,356,089

a Sequence length of the shortest contig at 50% of the total genome length.

Genomic DNA was extracted using an E.Z.N.A. bacterial DNA kit (Omega Bio-tek, Norcross, GA). Sequencing libraries were prepared using 1 ng of genomic DNA with a Nextera XT DNA sample prep kit (Illumina, Inc., San Diego, CA). PCR amplification was done in a Veriti 96-well thermal cycler machine (Thermo Fisher Scientific, Waltham, MA) using Nextera XT Index 1 primers (N7XX) from the Nextera XT index kit (FC131‐1001). Illumina paired-end sequencing was performed on a MiSeq platform using 2 × 250 paired-end sequencing chemistry. The raw data files were demultiplexed and converted to FASTQ files using Casava v.1.8.2. (Illumina, Inc., San Diego, CA). Quality control of the raw reads was performed using FastQC (Babraham Bioinformatics, Cambridge, MA). The reads were trimmed and assembled with BBDuk (DOE Joint Genome Institute, Walnut Creek, CA) and SPAdes (SPBU, St. Petersburg, Russia), respectively. The coverages of the assembled genomes were evaluated with BBMap (DOE Joint Genome Institute, Walnut Creek, CA).

The sizes of the genomes ranged between 4,766,150 and 5,104,082 bp, their coverage values ranged between 46.52× and 135.54×, and the numbers of contigs per assembly ranged between 62 and 174. Significant differences in the nucleotides and gene contents were detected between the isolates obtained from turkey environments and those obtained from chicken farm environments. Therefore, the availability of these genome sequences may facilitate understanding of *Salmonella* adaptation to different niches in poultry production (6).

### Data availability.

Draft genomic sequences have been deposited in the NCBI Sequence Read Archive (SRA accession number SRP126070) and NCBI GenBank (BioProject number PRJNA417775) and are listed in Table 1.
